# A mini review of inhaled beta 2 agonists in acute decompensated heart failure requiring respiratory support

**DOI:** 10.15761/pccm.1000161

**Published:** 2019-06-24

**Authors:** Nicholas Germano, Douglas Summerfield, Bruce Johnson

**Affiliations:** 1 Department of Internal Medicine, MercyOne North Iowa Medical Center, USA; 2 Department of Internal Medicine, Mayo Clinic, USA

**Keywords:** beta 2 agonists, acute decompensated heart failure, respiratory distress, respiratory failure, respiratory support

## Abstract

Acute decompensated heart failure (HF) results in over one million hospital admissions per year, many requiring invasive or noninvasive mechanical ventilation for respiratory/cardiovascular support. Inhaled beta-2 adrenergic receptor agonists have been shown to be effective at clearance of extravascular lung water in HF patients. However, studies done in the late 1990s and early 2000s, prior to standardization and wide adoption of guideline directed medical therapy for HF, suggested that inhaled beta-2 agonist use increased admissions for HF exacerbations as well as in-hospital mortality. One study even attempted to utilize intravenous Beta-2 agonists in Acute Respiratory Distress Syndrome patients, however the study was stopped prematurely due to an 11% increased mortality in the treatment group. More recently however, studies examining patients who have concurrent diagnoses of chronic obstructive pulmonary disease (COPD) and HF showed that beta-2 agonist therapy resulted in similar or better outcomes compared to controls. Likewise, in-vitro studies, animal models, and studies utilizing chronic heart failure patients treated with nebulized beta-2 agonists with no concurrent respiratory diagnosis had a therapeutic effect of treatment over controls. These studies have the advantage of being performed with the standardization of guideline directed HF medical therapy. In conclusion, while we continue to recommend the use of Beta-2 agonist therapy in patients with concurrent COPD and HF requiring respiratory support, further studies, preferably single or double blinded prospective trials, will need to be performed to determine whether Beta-2 agonist therapy offers morbidity and mortality benefits in patients with strictly acute decompensated heart failure requiring respiratory support.

## Introduction

Acute decompensation of heart failure remains one of the leading diagnoses for hospital admissions across the United States, with over one million admissions per year. A significant percentage of these cases are admitted to the intensive care unit (ICU) requiring emergent treatment due to respiratory distress or failure [1]. In HF patients, excess extravascular lung water (EVLW) leads to pulmonary edema which if left untreated or under-treated can lead to respiratory failure [2]. The current mainstay of treatment involves aggressive diuresis and optimization of medication, as well as rescue non-invasive or invasive mechanical ventilation for both respiratory and cardiovascular support [3,4]. Clearance of EVLW is one of the most important factors affecting positive outcomes in the inpatient setting [2].

Empiric therapy for respiratory distress, regardless of the etiology, in Emergency Departments and ICUs often utilizes Beta-2 agonists (B2A). Often these treatments continue even after diagnostic testing reveals fluid overload without concurrent COPD/Asthma exacerbation or pneumonia. There has been a variety of opinions, studies, and data that have shown outcomes both for and against such practice, of which this article will serve as a mini review of the points made over the last two decades.

## Beta-adrenergic receptors and their role in heart failure

The role of beta-adrenergic receptors in the pathogenesis of heart failure has evolved over the last two decades. Studies and reviews done by Madamanchi, Tong et al, and Lohse et al, utilizing isolated cardiomyocytes, in-vitro models, and mice knock out models, reached similar conclusions concerning beta-adrenergic receptor subtypes [5–7]. They found that the beta-1 subtype is the most prominent beta-adrenergic receptor in the heart and mainly responsible for positive chronotropic and inotropic effects of catecholamines, while the beta-2 subtype also increases cardiac function but does so through different pathways [5–7]. Both Tong and his colleagues, as well as Madamanchi, had data to support the theory that the beta-2 subtype is mostly cardioprotective while the beta-1 subtype has a much larger potential to be cardiotoxic and pro-apoptotic [5,6]. This, along with other studies of the time, helped reinforce the notion, and lead to the current thinking, that beta-1 receptors are more of the drivers of cardiotoxicity in heart failure than beta-2 receptors. This also explains the mechanism by which Beta-Blockers work to protect the heart [5–7].

However, in the last few years those findings are being challenged as more expanded studies have been completed. Data from a murine model study authored by Bernstein and colleagues, were able to elucidate the different signaling pathways that both major subtypes of beta-adrenergic receptors act on, and that those pathways are involved in both cardiac remodeling and contractility [8]. Within that same study there was some data supporting the idea that Beta-1 receptors are potentially cardiotoxic while Beta-2 receptors are cardioprotective, which on the surface looks to reinforce the original notion of the subtypes [8]. However this is not absolute, and it was found in their murine models that, based upon a range of different cardiac stressors involved, Beta-1 and Beta-2 receptors could change their status between both toxic and protective, and that they reacted differently on whether the stressor was acute vs chronic [8]. In the end, while cell survival and death appears to be regulated by the subtype of beta adrenergic receptor, the role that each subtype plays is dynamic and changes based upon stressors and the amount of time exposed to them, with this conclusion being reached also by Anthony Baker in his review of adrenergic signaling in heart failure [8,9]. Ultimately further studies will need to be done to map the effects of stressors and length of exposure to the effects it has on beta adrenergic receptor signaling and how it can dynamically change the state of the heart’s structure.

In regard to the lusitropic effects of beta 2 agonists, there has not been a wide range of studies on the subject, especially when compared to the well-established field of beta blocker therapy and their ability to slow myocardial relaxation. There is some data from the 1990s and early 2000s to support that isovolumic relaxation is accelerated when beta adrenergic agonists are added via intracoronary infusions of dobutamine in human patients [10]. Another study done by Kaumann et al. in the early 90s showed that catecholamine activation of beta-adrenergic receptors also hastens human ventricular relaxation and promotes the phosphorylation of various proteins throughout the system including phospholamban, troponin I, and C-protein [11]. Lastly beta 1 full and partial agonists, such as xamoterol, showed that beta adrenergic receptors have both inotropic activity and improvement in ventricular relaxation, while also increasing the atrial contribution to diastolic filling. There is also some data from animal models that show Beta-1 and Beta-2 agonists mediate relaxation of pulmonary vasculature [12,13]. As previously touched upon, the various beta-adrenergic receptors, and their effects, are modulated through multiple mechanisms and can change based on different stress states. Thus, more data will need to be collected on the effects of inhaled beta agonists on cardiac lusitropic effects as well as their effects on pulmonary vasculature in human patients.

## Beta-2 agonists and their isoforms

Human adrenaline causes bronchodilation and mast cell suppression, and it was these benefits that beta-2 agonists were designed to emulate through neuromediation. However, adrenaline exists naturally in our bodies as only R-adrenaline, while many beta-2 agonist inhalants/nebulizer treatments exist as racemic mixtures of R and S isomers [14]. Through extensive study it has been shown that while R-isomers exhibit the bronchodilation, anti-inflammatory and other clinically beneficial effects, S-isomers contribute to pro-inflammatory states, histamine release, exacerbate airway reactivity and bronchospasms, and is cleared far slower from the body than R-isomers [14,15]. In-fact, when S-albuterol is removed and simple levalbuterol is given, the needed bronchodilation and other clinical benefits are achieved at one-quarter to one-half of the dose of the racemic combination, and there is a marked reduction in side effects as well [14,16].

## Beta-2 agonists and extravascular lung water clearance

Patients with heart failure with reduced ejection fraction (HFrEF), even when stable on guideline directed medal therapy (GDMT), have elevated EVLW at baseline [17]. When comparing HFrEF patients and healthy controls, B2A has also been shown to decrease the EVLW [17]. A more in-depth analysis has revealed that heart failure patients have structurally similar lungs to healthy ones, specifically airway wall thickness and luminal cross-sectional area, which is being maintained between healthy and CHF groups [17]. Furthermore, in stable patients, B2A nebulization treatments increases clearance of EVLW but do not fundamentally alter lung structure, which theoretically should help stabilize the patient faster by improving diuresis [17].

The ability of beta-2 agonists to clear fluid through the alveolar epithelium is postulated to be through two main mechanisms. The first revolves around stimulation of lymphatic based beta-2 adrenergic receptors. This induces active phasic contraction of lymphatic ducts, as well as thoracic lymphatic dilation helping to clear fluid found in perivascular spaces and hilar lymph nodes [18]. The second mechanism is due to its association with the amiloride-sensitive sodium channels as well as with the Sodium-Potassium ATPase pump (Na+/K+-ATPase pump) [19]. Both alveolar type I and II cells have amiloride-sensitive sodium channels on their apical membranes, and sodium is then extruded from the basolateral surface by the Na+/K+ ATPase pump. Alveolar type 1 cells, comprising roughly 90% of alveolar surface area, also express the alpha-2 isoform if the amiloride-sensitive sodium channels, playing a major role in active sodium transport, as well as Na+/K+-ATPase pumps [19]. Water follows the osmotic gradient passively, absorbed through aquaporin channels 1 and 5, which are expressed on the microvascular endothelium and type 1 alveolar cells in submucosal gland acinar cells respectively [19]. Due to their resistance to injury, type II alveolar cells are mainly responsible for edema clearance when lung injury is present, through the above mechanisms. Beta-2 adrenergic agonists module the expression of these two pumps in the short run, however long-term stimulation desensitizes the pathway, reducing the amount of fluid clearance during acute stress [19].

## Beta-2 agonists prior to heart failure goal directed medical therapy

Some studies completed in the 1990s and early 2000s support the notion that, despite physiologic evidence of improved EVLW clearance, there is increased in-hospital mortality rates in B2A therapy groups when compared to controls [20]. One multi-centered trial utilized intravenous B2A treatments on Acute Respiratory Distress (ARDS) patients, however it was stopped prematurely as the treatment group had an 11% increased mortality rate above the control [21]. It should be noted this trial used IV B2A instead of inhaled therapies, and also lacked extensive multi-variable analysis and propensity scoring. A study completed by Au and Colleagues, done in the early 2000s, recruited over 1500 subjects and showed that those patients with left ventricular systolic dysfunction who were on chronic inhaled beta-agonists were at increased risk for heart failure hospitalization and all-cause mortality [20]. This was further reinforced, albeit with a smaller patient population, by another study from the same authors in 2004 which showed that while chronic B2A therapy did not increase the incidence of chronic heart failure, there was some association with increased risk of hospitalization for CHF for those with a prior CHF diagnosis [22,23]. While these studies looked at chronic B2A therapy, not acute treatment upon hospitalization, they did take into account and adjust for other confounding conditions, such as age, diabetes, hypertension, acute coronary syndromes, cardiovascular disease, and alcohol abuse [20]. However, they were prior to our current standardized and wide spread adoption of GDMT for heart failure, which is important considering its emphasis on beta-blocker use as a mainstay of modern day treatment.

## Guideline directed medical therapy in heart failure

While there have been focused updates since, the American College of Cardiology issued its overarching recommendations in 2013, with the class of drugs being used for the treatment of heart failure being tied to the stages of the disease [24]. Guideline directed medical therapy utilizes lifestyle changes, statins, but most importantly ACEI/ARBs, beta blockers, aldosterone antagonists, diuretics, and if need be, implantable devices to control symptoms and progression as well as to treat exacerbations [24]. The guidelines call for stage A patients (those at high risk for heart failure but no structural disease or symptoms) to be started on an ACEI or ARB, and as the disease process progresses increasing amounts of pharmacotherapy are added [24]. Stage B, which shows structural changes to the heart but with the patient lacking signs or symptoms of heart failure, calls for the addition of beta-blockers. Aldosterone antagonists are added in Stage C (Stage B plus prior or current symptoms) [24]. The formalization and widespread adoption of guideline directed medical therapy for heart failure has seen a significant improvement in mortality and morbidity, compared to the 1990s and early 2000s [24]. Use of beta-blockers in particular have shown a remarkable reduction in all-cause mortality in heart-failure patients, with some studies showing upwards of 30% or greater mortality reduction [24,25].

## Beta-2 agonists in the era of goal directed therapy

A recent PubMed literature search using the terms Beta 2 agonists, heart failure, and respiratory support revealed no single or double blinded prospective study on the use of beta-2 agonists in heart failure patients requiring rescue BiPAP or, more importantly, requiring invasive mechanical ventilation. There has been some research on Beta-2 agonists on people with concurrent heart failure and COPD, who present with respiratory distress/failure secondary to their COPD. In these studies patients performed as well as or superior to the control groups, helping to demonstrate the safety and effectiveness of the treatments within the context of the concurrent conditions [26–28]. One postulated explanation for the discrepancies in outcomes between earlier trials and these later ones, is that trials of B2A treatment in strictly heart failure patients, who are now on goal directed therapy, has not been thoroughly explored. The tachycardia and changes in hemodynamic status that can result from B2A therapy in trials done in the late 90s and early 2000s could result in more in-hospital complications, such as ischemic events, that may not be present to the same extent today.

More recently, when considering heart failure secondary to dilated cardiomyopathy, in-vitro and animal studies have shown that the combination of B2A agonists and B1-blocker is more effective than beta-blocker alone and is as effective as B1-Blocker and ACEI with respect to treatment [29,30]. A study done in the outpatient setting sought to evaluate the effectiveness of inhaled bronchodilators on pulmonary function and dyspnea in patients with heart failure. This study excluded COPD and Asthma patients and showed the potential of B2A in improving pulmonary function compared to the control groups [30–32]. While these studies did not directly look at outcomes for acute B2A treatment in respiratory support dependent heart failure exacerbations, the data extrapolated from these studies, as well as the physiological studies previously done, suggest a benefit from B2A therapy being utilized in such a setting. However, further studies will be needed to evaluate the true efficacy of such treatments.

## Potential drug-drug interactions and side effects

Beta-2 agonist induced hypokalemia, or exacerbation of hypokalemic states, is an unfortunate side effect of therapy, and something to be aware of when utilizing them in acute heart failure exacerbations. The most common diuretics used in hospitals today, including furosemide, torsemide, and bumetanide can all cause hypokalemia, and in combination with beta-2 agonists, which cause the shifting of serum potassium into cells, can cause worsening of serum potassium and magnesium levels resulting in cardiac arrythmias, and therefore caution should be used when administering concurrently, especially in high doses of either medication [33].

Currently there are package inserts/caution against the use of sustained albuterol treatment and Monoamine Oxidase Inhibitors, Tricyclic Antidepressants, and Einezolid, likely due to the inhibition of monoamine oxidase which, through deamination and methylation, helps to terminate the action of beta-2 agonists (and adrenaline) [34]. There is concern for serotonin syndrome, blood pressure instability, arrythmias, and palpitations when used concurrently [33]. However, there have been some studies to show that, in regard to linezolid, there is very low occurrence when compared to control groups [35].

## Conclusion

The existing data and trials support the use of inhaled beta-2 agonist treatment for patients in respiratory distress who possess concurrent COPD and heart failure. However, based on data from the last twenty years, there are arguments for and against treating patients with strictly acute decompensated heart failure requiring respiratory support on bronchodilators. Most concerning is the potential for increasing mortality with such a practice. However, as discussed above, the increased risk of harm may be reduced in the current age of goal directed medical therapy and the decompensated heart failure patient on appropriate GDMT may theoretically benefit from inhaled beta-2 agonists. With the limited in vivo studies, however, we currently do not advise this as standard practice. However, given the empiric treatment with beta-2 agonists in patients with respiratory distress, with the potential for harm, or benefit in the GDMT treated patients, it is our recommendation that further studies be performed to further elucidate the answers.
